# Prevalence of American foulbrood in asymptomatic apiaries of Kurdistan, Iran

**DOI:** 10.14202/vetworld.2018.281-285

**Published:** 2018-03-05

**Authors:** M. Khezri, M. Moharrami, H. Modirrousta, M. Torkaman, B. Rokhzad, H. Khanbabaie

**Affiliations:** 1 Department of Veterinary Research, Kurdistan Agricultural and Natural Resources Research Center, AREEO, Sanandaj, Iran; 2 Department of Honey Bee, Silk Worm and Wildlife Research Diseases, Razi Vaccine and Serum Research Institute, Karaj, Iran

**Keywords:** American foulbrood, honeybee, Kurdistan province, *Paenibacillus larvae* subsp*. larvae*, polymerase chain reaction

## Abstract

**Aim:**

*Paenibacillus larvae* subsp. *larvae* is the etiological agent of American foulbrood (AFB), the most virulent bacterial disease of honey bee brood worldwide. In many countries, AFB is a notifiable disease since it is highly contagious, in most cases incurable, and able to kill affected colonies. The aim of this study was to determine the prevalence of *P. larvae* subsp*. larvae* in Kurdistan province apiaries by polymerase chain reaction (PCR) technique.

**Materials and Methods:**

A total of 100 samples were randomly purchased from apiaries in Kurdistan, Iran. Apiaries were randomly sampled in accordance with the instructions of the veterinary organization from different provinces and were tested using PCR method and an exclusive primer of 16S rRNA for the presence of *P. larvae* subsp*. larvae*.

**Results:**

The results of this study indicated a low level of contamination with *P. larvae* subsp*. larvae* in the Kurdistan province. The number of positive samples obtained by PCR was 2%.

**Conclusion:**

Therefore, monitoring programs for this honeybee disease in Kurdistan should be developed and implemented to ensure that it is detected early and managed.

## Introduction

Honey bees and larvae are subjected to a wide range of microorganisms. Considering the economic importance of the beekeeping in Iran and the role of honey bee products in various uses, it is essential to be aware of bee diseases and timely diagnosis. American foulbrood (AFB) is one of the most dangerous and important diseases of brood honey bees which affect larvae of *Apis mellifer*a honey bee [1].

This disease is caused by the Gram-positive bacteria with spores called *Paenibacillus larva*e under the *larva*e species. Spores can survive in the environment 35-50 years [2]. Spores are resistant to drought, high temperatures (100°C for more than 5 min), and ultraviolet (UV) light. Furthermore, in contact with conventional disinfectants, such as formaldehyde solution, they can survive 10% more than 5 h [3]. This makes the control of the disease difficult because human activity can spread the disease over long distances and previously dormant strains may cause an outbreak several years after the original outbreak [4]. The presence of *P. larva*e subs*p. larva*e spore in the hive indicates that the hive is contaminated, and as soon as the condition is to be available for growth, the spores germinate and cause disease [2]. Spores attack honey bees in the larval stage (usually during the first 24-36 h of life) [5]. Larvae with more than 2 days are more resistant to infection, but in very young larvae, 10 spores or less effectively cause the disease [6]. Spores germination occurs at 6.6 pH and 36-37°C temperatures under the conditions of 5-10% microaerophilic CO_2_. Spores develop in the middle intestine, almost 1 day after swallowing by the larva. Tube cells are not able to proliferate in the larvae intestine, and thus, by the help of the flagella, they penetrate from the epithelium into the body cavity and proliferate in the hemolymph. Larvae die due to a systemic bacterial infection [7]. After death, the larvae, which are usually white, change the color to the brown mass and then disintegrate and place on the cell floor. After degradation of the body, with increasing viscosity of the body, the larvae gradually stick to the bottom and wall of the cell as a scale after a short period of time [5]. The host-specific structure in disease transmission is important because the high density of colonies and beekeeping in one area promotes and spreads the disease factor [8]. The ultimate diagnosis of the disease is based on laboratory methods, cultivation, and isolation of the bacteria causing the disease. The cause of this disease is late and hard growing, and its detection requires up to 2 weeks using cultivation methods and biochemical diagnostic kits. However, using accurate molecular detection methods such as polymerase chain reaction (PCR), infections can be detected much less frequently in the colony before the observing clinical signs, and the disease can be controlled by continuous analysis of the infection reservoir (worker bees) [9]. To confirm a disease suspicion or to monitor the prevalence of *P. larva*e subs*p. larva*e, various products from the honeybee hive (e.g., honey, bees, wax, pollen, and debris) can be sampled for laboratory analysis [10]. In several recent studies in different parts of the world, molecular detection methods have been used to detect *P. larva*e subs*p. larva*e [11-15]. Primers derived from the 16S rDNA region can be used to perform PCR. This marker is widely used to examine the occurrence and spread of bacteria in various samples [16].

The aim of this study was to determine the prevalence rate of *P. larva*e subsp*. larva*e in the apiary of Kurdistan province and its confirmation by molecular and standardized PCR diagnostic method using adult bee samples.

## Materials and Methods

### Ethical approval

This research was approved by the Science and Research Committee of Razi Vaccine and Serum Research Institute, Karaj, Iran. The collection of clinical samples only required the owner´s approval as mentioned in Materials and Methods.

### Sampling

The simple random method was used to select the samples. In this method, using the formula 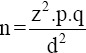 and the correction coefficient SPC, 100 apiaries of Kurdistan province were selected randomly, and according to the instructions of the veterinary organization [17], the required sample was taken from 5% of the colonies in each apiary and transferred to the Honey Bee, Silk Worm and Wildlife Research Diseases Department of Razi Vaccine and Serum Research Institute as soon as possible.

### Preparation method of honey bee sample to extract spores

A total of 20 bees were crushed in 10 ml sterilized distilled water, and a homogeneous solution was prepared and purified using gauze and subsequently centrifuged at 1800g for 5 min. Then, the centrifuge solution recentrifuged at 6000g for 30 min and the resulting precipitate which probably contained spores was suspended for separating DNA of 1 ml of sterile distilled water [18].

### The method of DNA extraction from spore-containing samples

The suspensions prepared in 6000g were centrifuged for 30 min, and then, the spore walls of the samples were destroyed by enzymatic method and their DNA was used for PCR testing [18].

### PCR

To do PCR testing, primers that were designed by De Graaf *et al*. [19] based on the sequence of the 16S rDNA gene of the bacterium were used. Primer sequences were determined based on regions that contain one base difference between *P. larvae* subsp. *larvae* and *P. larvae* subsp. *pulvifaciens* (AY 030080) at the 3’ ends of the sequences. The expected amplification fragment size was about 700 bp.

F: (5’-TCAGTTATAGGCCAGAAAGC-3’),

R: (5’-CGAGCGGACCTTGTGTTTCC-3’).

The PCR reaction was performed with a final volume of 25 µl, 2.5 µl, 10×PCR buffer, 0.5 µl of 10 mM dNTP mix solution, 1 µl of a concentration of 10 µm of each primer, Taq (1U), 2 µl of 25 mM MgCl_2_ solution, and 1µl of extracted DNA and distilled water was used. PCR was performed in an Eppendorf gradient thermocycler with the condition of initial denaturation at 95°C for 1 min and the next 30 cycles as denaturation at 95°C for 1 min, annealing at 55°C for 30s, extending with a temperature of 72°C for 1 min, and a final extending cycle at a temperature of 72°C for 5 min [20]. 10µl of PCR product was mixed with 2µl of buffer loading solution and added to 0.8% agarose gel wells containing ethidium bromide. For this, 1 kb of DNA marker was used. After the electrophoresis time was completed, the gel was put on the UV-trans illuminator device to study and take pictures [21].

## Results

**Figure-1:**
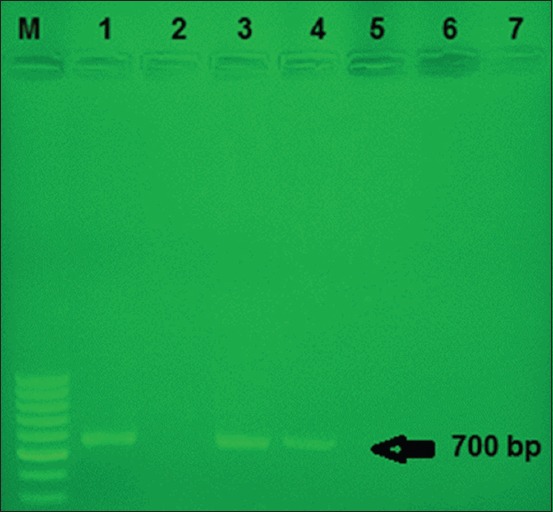
Agarose gel electrophoresis of polymerase chain reaction products using specific primers and DNA from different *Paenibacillus larvae* subsp*. larvae* isolate as a template. M: 1 kb marker, 1: Positive control, 2: Negative control, 3-4: Positive samples, 5-7: Negative samples.

PCR test was performed on 100 samples that collected randomly from the Kurdistan province and the results showed that two samples were positive (Figure-1), and to confirm the results, suspension of the two prototypes of two positive samples was cultivated on two plates containing MYPGP agar medium, each of which was 200 µl. Plates were incubated under microaerophilic conditions (5-10% CO_2_). A total of 3-5 bacterial colonies from each plate were used for confirmatory tests and repeated PCR, and the results obtained at this stage confirmed the preliminary results.

## Discussion

In the past, the diagnosis of AFB disease was largely based on the cultivation and isolation of the disease factor, and the discriminatory diagnosis of the disease from other bacteria was very time-consuming and based on morphology and biochemical patterns. This subject attracted the attention of many scholars to develop more rapid methods to diagnose the disease. In this regard, molecular techniques provide the possibility of detection within a very short time. In 1999, for the first time using PCR, grown colonies of *P. larvae* subsp*. larvae* were detected in a semi-specific culture medium [12]. In 2001, a study was conducted on *P. larvae* subsp*. larvae* isolate related to the honey and larvae, and it was reported that the sensitivity of PCR was very specific using the primer *P. larvae* subsp*. larvae* and created no cross-reaction with other similar bacteria [15]. In several other studies, the PCR method has been used to replicate the 16S rRNA gene for detecting *P. larvae* subsp*. larvae* [11,20]. In this study, using PCR method, bee samples of Kurdistan Province apiaries were screened in terms of the prevalence of *P. larvae* subsp*. larvae* bacterial spore. In this research, a PCR diagnostic technique was used for rapid detection of *P. larvae* subsp*. larvae* spores isolated from nurse bees. Molecular method has been shown to be a useful tool for the diagnosis of AFB as it offers the advantages of fast, sensitive and reliable diagnosis. The results of this study showed that of 100 specimens collected from the province, two specimens were positive in the PCR test (2%), which indicates a relatively low rate of AFB disease in the apiaries of the province. To detect spores of AFB disease, many colony-like parts and products such as larvae, adult bees, pollen, honey, royal jelly, and wax are required to be sampled [1]. Lindström and Fries [22] have shown that sampling of adult bees in screening studies of AFB disease in one region is the most appropriate type of sample. Disease diagnosis using samples of honey and adult bees has a higher prognostic value compared to the detection of the bacteria in wax, pollen, and debris samples [23,24]. Regular disease monitoring is important because if AFB is not detected and treated, it will lead to the loss of the infected hive and serve as a major source for infections to neighboring colonies [25]. Ritter [26] found that timely diagnosis of the prevalence of AFB disease through testing honey and wax in honey which is available in the hive is one of the ways that are currently being applied globally. Due to the nature of the disease and the difficulty of treating with honey and wax test, the disease becomes evident, and he added that in testing 700 specimens of honey produced outside Europe, about 98% are contaminated with *P. larvae* subsp*. larvae* and the report further states that of the European honey production, 62% are contaminated with spores, and by testing 420 specimens, 70% of honey bees are contaminated with spores so that in 1 g of wax, 1000 spores have been observed. The research conducted in the Hatay and Adana Provinces of Turkey has shown that the prevalence of AFB disease was 29% [27]. The prevalence of AFB disease of honeybee in north-west Pakistan was 37.30% [28]. Haddad *et al*. [29] reported that the total of 57 (honey brood and brood nest honey) from different regions of Jordan was inspected to carry *P. larvae* spores with 35%. In another study, specimens of honey produced in Tehran Province were screened by PCR to determine the contamination rate of *P. larvae* subsp*. larvae* spores, and it was found that 25.6% of the tested specimens were contaminated with *P. larvae* subsp*. larvae* spores indicating a relatively high prevalence of this disease in the apiaries of Tehran Province [30]. Yusefkhani and Lotfi [31] reported that the rate of contamination of hives in East Azerbaijan Province with AFB disease was 5.8%. During 2010-2011, in testing 100 apiaries of West Azerbaijan Province, it was revealed that 97 apiaries had no contamination with *P. larvae* subsp*. larvae* and the bacterium causing AFB disease was isolated from larvae, wax, and honey sample, as well as two samples of worker bees [32]. The research carried out in Lorestan Province on the honey bee larvae by PCR method showed that the contamination rate of the tested hives was 13% [33]. The PCR method is considered as a new strategy for screening the factor of an important and harmful AFB disease in Iran. By the help of this strategy, it is easy to evaluate a large number of specimens at a shorter time and also lower cost and to judge correctly about the condition of disease or contamination and possible epidemics in the region and the country.

## Conclusion

The distribution of *P. larvae* subsp*. larvae* spores in few samples of the Kurdistan Province showed a clear pattern and may provide useful data for the strategy of control and non-spreading of AFB.

## Authors’ Contributions

MK designed the study. MK, BR, and HK collected and processed the samples for isolation and identification of bacteria. MK, MM, HM, and MT were done PCR and electrophoresis. MK and MT interpreted the results and analyzed the data. MK prepared the manuscript. All authors read and approved the final manuscript.
